# Interventions to support the mental health of family carers of children with brain injury in low and middle income countries: a scoping review

**DOI:** 10.3389/fresc.2024.1405674

**Published:** 2024-08-29

**Authors:** M. A. Linden, R. Leonard, L. Ewing-Cobbs, K. C. Davis, L. Schrieff-Brown

**Affiliations:** ^1^ School of Nursing & Midwifery, Queen’s University Belfast, Belfast, United Kingdom; ^2^ Department of Pediatrics, UTHealth Houston, McGovern Medical School, Houston, TX, United States; ^3^ Department of Pediatrics, Baylor College of Medicine, Houston, TX, United States; ^4^ Department of Psychology, University of Cape Town, Cape Town, South Africa

**Keywords:** scoping review, low and middle income countries, brain injury, families, carers

## Abstract

**Aim:**

To review the international evidence base on interventions to support the mental health of family carers of children with brain injuries in low and middle income countries (LMIC).

**Methods:**

Searches were conducted with five electronic databases (Pubmed, Web of Science, Embase, PsycINFO, CINAHL) using search terms related to “family carers”, “brain injury”, “children” and “low and middle income countries”. Studies were independently screened using predetermined eligibility criteria by two authors. Data were extracted from included studies using standardised data extraction and quality appraisal tools. These data were then subjected to narrative synthesis. The Preferred Reporting Items for Systematic reviews and Meta-Analyses (PRISMA) guidelines were used to govern the review process.

**Findings:**

One study met our inclusion criteria and described an acquired brain injury called nodding syndrome which occurs in Sub-Saharan Africa. The study was conducted in Ghana and provided group-based psychotherapy to carers and their children. As such we found no study which sought to solely support the mental health of family carers.

**Conclusions:**

There has been a lack of focus in the literature on the mental health of family carers of children with brain injuries in LMIC. Considering the vital importance of caregivers in supporting their children it is imperative that service providers and researchers devise programmes to better meet their needs. The mental health of family carers should be better supported to improve their overall wellbeing, which will in turn improve the wellbeing of their children.

## 1 Introduction

Acquired brain injury (ABI) is a major global health concern. Worldwide, millions of children and youth are affected by ABI each year (1, 2). For this review, we define ABI as injuries to the brain arising after birth. Although traumatic brain injury (TBI) is the most common aetiology, the broader category of ABI includes stroke, infection, tumours and cancer, hypoxic-ischemic insult, epilepsy, and diseases of the brain. Much of the research reviewed in this article is based on TBI, which is recognized as the most common global external cause of morbidity and mortality. As with other ABIs, the burden of disease is higher in countries classified as being low and middle-income (LMIC) relative to countries with greater resources (3). The burden of disease in LMIC is higher for several reasons, including elevated incidence due to population increases, as well as global disparities in pathology, prevention initiatives, access to care and rehabilitation, and clinical guidelines that can be used in settings with limited resources (3). ABI sustained during childhood is associated with particular challenges due to the high incidence and cumulative prevalence, as well as the years of life affected. Particularly following more severe brain injuries, acute symptoms may evolve into chronic health conditions requiring long-term or lifetime care (4).

Childhood ABI impacts the lives of all family members. Following injury or diagnosis of ABI, families report increases in uncertainty, stress, anxiety, and depression that often persist (5–7). Burden is increased in families whose children experienced more severe injury, children having continuing medical and/or behavioural health problems, and in parents reporting unmet health care needs (8–10). These needs shift over time from primarily physical to cognitive and socioemotional needs, especially in families with greater socioeconomic stress (11, 12). Even in resourced settings, parents of children with ABI noted unmet needs in several areas, including providing information, facilitating transitions, and addressing emotional and psychological health challenges (13–15). Lower parental socio-economic status and increased anxiety have been associated with more child behaviour problems and reduced quality of life in children with mild TBI (16). Psychosocial difficulties in children following mild TBI have been found to be predicted by parental depression, anxiety and socioeconomic status four years post injury (17). The impact of parental mental health and well-being on child and family outcomes cannot be underestimated (17). Indeed, practice recommendations encourage a family-centred approach to service provision as an approach to supporting the needs of children, parents, and families (18, 19).

Better family functioning prior to diagnosis or injury is a consistent predictor of better child outcomes. As this relationship is often bidirectional (20), supporting and empowering parents and families is likely to have a positive impact on the family system and child outcomes. Recognizing the importance of the family, Braga and colleagues developed a family-based method of rehabilitation delivery and integrated parents into the care team, resulting in improved child outcomes in a limited resource setting (21). Similarly, parent-based interventions have shown positive impacts on both child behaviour and parent coping after ABI (22). Two US based studies have shown improvements in the behaviour of children who sustained moderate to severe TBI following participation in an online parenting skills programme (23), and the alleviation of parental distress following a counsellor-assisted problem solving intervention for those from low socio-economic backgrounds (24). An Australian pilot study exploring psychoeducation for family carers in managing challenging behaviours following TBI demonstrated acceptability, but suggested it would most benefit those at the start of the caregiving journey (25).

There has been a lack of research focusing on how LMIC provide support to families following ABI. This review sought to explore what interventions existed to support the mental health of family carers of children with ABI in LMIC.

## 2 Methods

### 2.1 Search strategy

The search strategy was developed with the assistance of a specialist subject librarian, based on the authors’ experience of the area and through review of existing research in the field. The strategy was built around five key areas which reflected the research question. These included “brain injury”, “families”, “children”, “low and middle income countries” and “mental health” (see Table 1). Searches were conducted in November 2023.

**Table 1:** Search terms.

Key terms	Brain injury	Families	Children	Low and middle income countries	Mental health
Search terms	“Traumatic brain injury” OR TBI OR “Acquired brain injury” OR ABI OR “Brain Injur*” OR “Head injur*” OR “Craniocerebral Trauma” OR “Cerebrovascular Trauma” OR Brain OR “Brain Swelling” OR “Cerebral Edema” OR “Glasgow Coma Scale” OR “Glasgow Outcome Scale” OR Unconsciousness OR Pneumocephalus OR Epilepsy OR Post-Traumatic OR “Cerebral Haemorrhage” OR “Brain Damage”	Famil* OR “family members” OR “family unit” OR “family system” OR “family network” OR “family relations” OR “family carers” OR carer* OR caregiver* OR “family caregiver” OR “family caregivers” OR “adult-children” OR children OR parent* OR grandparent* OR grandchild*	child* OR adolescen* OR “young people” OR “young adult”	“Developing Countries” OR “developing countr*” OR “under developed countr*” OR lmic* OR “less developed” OR “low income” OR “lower income” OR “low and middle income” OR “low middle income” OR “resource poor” OR “resource constrained” OR “low resource” OR “limited resource*” OR “resource limited”	Mental health OR mental disorder* OR “mentally ill persons” OR “substance related disorder*” OR “alcohol related disorder*” OR “anxiety disorder*” OR “anxiety neuroses” OR “neurotic anxiety states” OR “anxiety neurose*” OR anxiety OR “separation anxiety” OR panic OR “panic disorder” OR agoraphobia OR “obsessive behaviour” OR “obsessive-compulsive disorder” OR OCD OR “phobic disorders” OR “depressive disorder*” OR depression OR “involutional depression” OR “seasonal affective disorder” OR “eating disorder” OR “anorexia nervosa”

### 2.2 Information sources

Five electronic databases were systematically searched (PubMed, Web of Science, Embase, PsycINFO and CINAHL) using the search terms shown in Table 1. Databases were selected due to their inclusion of a broad range of international literature and diverse discipline focus.

### 2.3 Eligibility criteria

We included only peer reviewed empirical publications and excluded books, magazine articles, abstracts and systematic reviews. Included publications had to address our research question and so related to supporting the mental health of family carers of children with brain injury in LMIC. LMIC were defined as those included on the Development Assistance Committee list for Official Development Assistance (26). Studies which focused on training family carers to support their children were excluded. No exclusions were placed on study designs. Due to resource constraints, we limited included studies to those published in the English language.

### 2.4 Selection process

**Figure 1:**
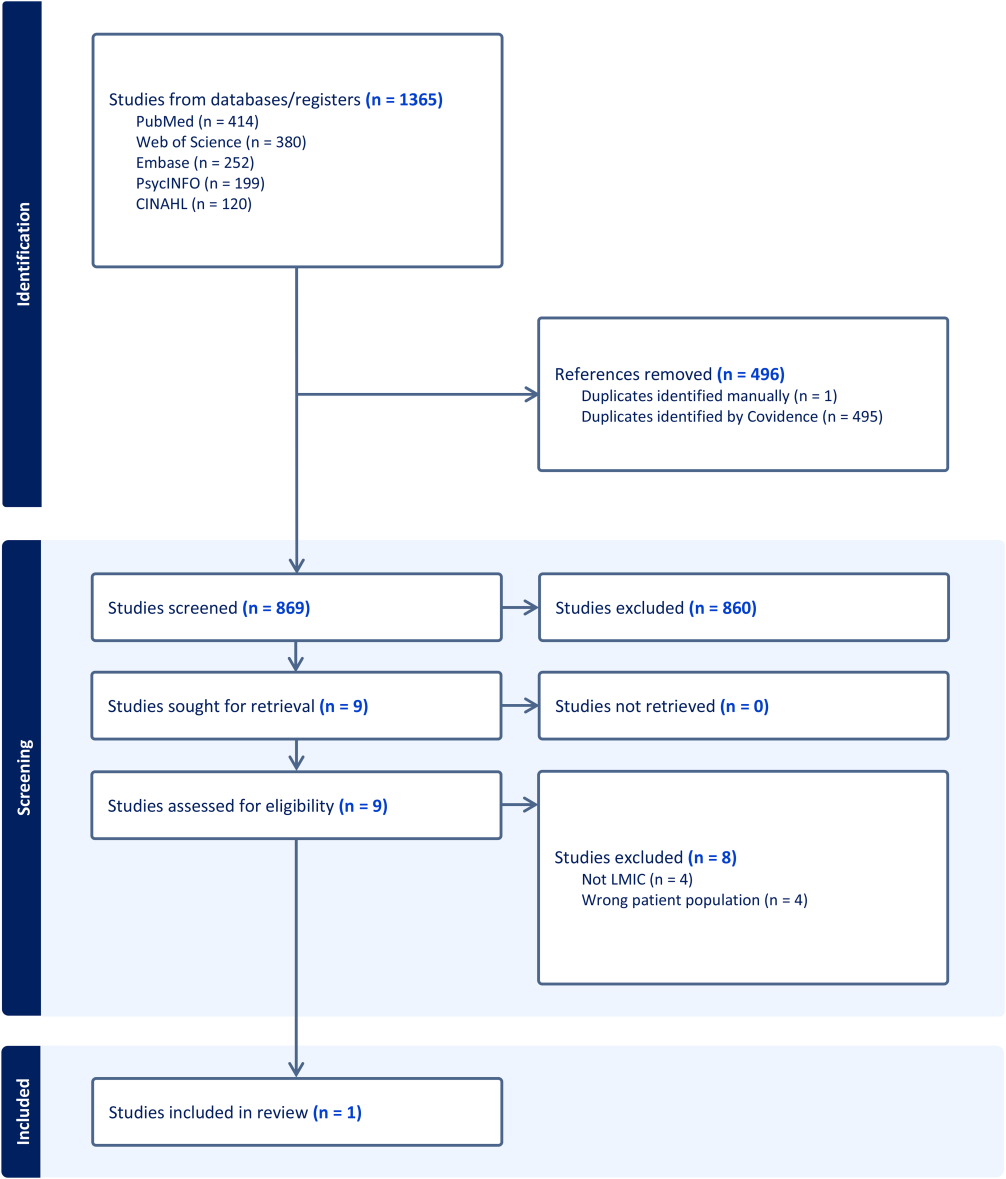
PRISMA flowchart of selection process.

Database searches revealed a total of 1,365 records from, PubMed (*n* = 414), Web of Science (*n* = 380), Embase (*n* = 252), PsycINFO (*n* = 199) and CINAHL (*n* = 120). These were imported into Covidence (27), a web-based collaboration tool which supports the conduct of systematic reviews, and duplicates were removed (*n* = 496). The titles and abstracts of the remaining records (*n* = 869) were independently reviewed by two authors who applied the above eligibility criteria. We excluded *n* = 860 records and retrieved nine papers for full text screening. Any conflicts were discussed and agreed upon by consensus. Following review of the full text papers a further eight were excluded. Reasons for exclusion related to the research being conducted in a high income country (*n* = 4) and incorrect population (*n* = 4) i.e., a focus on family carers supporting their children. Therefore, one paper was included in this review. See Figure 1 for the Preferred Reporting Items for Systematic reviews and Meta-Analyses (PRISMA) (28) flowchart.

### 2.5 Data extraction

Data from the eligible study was independently extracted by two authors. Reviewers employed a standardised data extraction tool to ensure consistency. Any disagreements were resolved through discussion until consensus had been reached. Data extracted included author name, date of publication, country of origin, type of brain injury, aim, study design, participants, measures and key findings. The extracted data can be found in Table 2.

**Table 2:** Characteristics of papers included in this review.

Authors Name, Year of Publication, Country of origin Type of ABI	Aim/Hypothesis	Design	Participants	Measurement used. If Qual, questions asked.	Key findings & Recommendations	Quality appraisal (MMAT)
Mutamba et al. (2018a) (31) Uganda ABI (nodding syndrome)	To evaluate the effectiveness of a group interpersonal psychotherapy (IPT-G) intervention, when delivered by community health workers in a low-resource government health system in Uganda	Non-randomized trial Participants were divided into two groups. 1) interpersonal group psychotherapy, 2) usual care.	Group 1 = *n* = 73, female = 59 (80.8%), age = 43.4 (9.0), formal education = 39 (53.4%) Group 2 = *n* = 69, female 50 (72.5%), age = 41.8 (12.3), formal education = 44 (63.8%)	Depression, generalized anxiety disorder PTSD and suicide risk assessment MINI Neuropsychiatric interview; Psychological distress assessed using the Self Report Questionnaire (SRQ-20); Functional impairment assessed via the Assessment of Functioning questionnaire (locally developed tool); Alcohol Use Disorders Identification Test (AUDIT); Multidimensional Scale of Perceived Social Support (MSPSS); Perceived stigma assessed via the Devaluation of Consumer Scale; Number and history of war-related traumatic events assessed using the traumatic events checklist (locally developed tool).	Caregivers who received the intervention had a significantly greater reduction in the risk of depression from baseline to 1 month [risk ratio (RR) 0.25, 95% confidence interval (CI) 0.10–0.62] and 6 months (RR 0.33, 95% CI 0.11–0.95) post-intervention compared with caregivers in group 2 (control). IPT-G delivered within a low-resource health system is an effective Psychological Treatment for common mental health problems in caregivers of children with a severe neuropsychiatric condition and has psychological benefits for the children as well.	6/7

### 2.6 Quality appraisal

Quality of the included study was assessed using the Mixed Methods Appraisal Tool (MMAT) (29). The MMAT asks reviewers to answer two screening questions concerning a clear research question and whether data collection can address this question. These are followed by the reviewer choosing between one of five possible study designs, each containing five questions. Responses to these questions are recorded as “yes”, “no” and “can't tell”. Included studies can be rated out of seven with a “yes” indicating one point. Table 3 displays the quality appraisal for the study included in this review.

**Table 3:** Quality assessment using the MMAT.

First author & year	Q1	Q2	Q3	Q4	Q5	Q6	Q7	Explanation
Mutamba et al. (2018a) (31)	Y	Y	Y	Y	N	Y	Y	Results for war trauma checklist not reported

Q1 Are there clear research questions?; Q2 Do the collected data allow to address the research questions?; Q3 Are the participants representative of the target population?; Q4 Are measurements appropriate regarding both the outcome and intervention (or exposure)?; Q5 Are there complete outcome data?; Q6 Are the confounders accounted for in the design and analysis?; Q7 During the study period, is the intervention administered (or exposure occurred) as intended?

### 2.7 Data analysis

As there was only one study included in this review it was not possible to pool data for meta-analysis. Instead, we sought to employ narrative synthesis (30) to examine key themes identified within the paper and contrasted these with the eight papers excluded at full text review to identify areas of best practice and future research. It was reasoned that contrasting the included study with excluded carer-based interventions from high income countries, or with studies which focused on carers as a support for their children, might provide important comparative data.

## 3 Results

### 3.1 Characteristics of included studies

Mutamba et al. (31) recruited 142 family carers and child dyads for their study. Participants were placed into the intervention (*n* = 73, group based psychotherapy) and control (*n* = 69, usual care) conditions with recourse to randomisation procedures. The authors failed to describe what they meant by usual care. The research was conducted in Uganda which is considered as a lower middle income country by the DAC list of ODA recipients. Family carers were supporting their children with brain injuries which were acquired through nodding syndrome, a condition which is unique to Sub-Saharan Africa. The precise aetiology of nodding syndrome is unknown, however, suggested mechanisms of injury have included infection by Onchocerca volvulus (parasitic worm), munitions-related neurotoxins, food-related toxins and nutritional deficiencies (32).

### 3.2 Quality of included studies

Quality of the Mutamba et al.'s (31) study was assessed via the MMAT (29) which was rated out of seven. Higher scores indicate an article has greater methodological quality. Mutamaba et al. (31) scored 6 out of 7 indicating high quality (see Table 3 for quality assessment). This paper lost a point due to a failure to fully report on all measurements collected. Specifically, the authors did not report complete outcomes relating to the number and history of war-related traumatic events.

### 3.3 Narrative synthesis

As our review included a single paper it was not possible to conduct a traditional narrative synthesis. As such we sought to compare characteristics of Mutamba et al. (31) with the eight papers excluded at full text screening. Papers excluded at this stage included Mutamba et al. (33), Bass et al. (34), Wade et al. (35), Petranovich et al. (24), Raju et al. (36), Raj et al. (37), Carlo et al. (38) and Robertson et al. (39).

### 3.4 Place and study design

Excluded papers referred to work which was conducted in Australia (39), India (36), India, Pakistan, and Zambia (38), Uganda (33, 40), and the USA (24, 35, 37). Our included study was also conducted in Uganda. Of those conducted in LMIC, a case report focused on medical and psychiatric social workers in India and how they might support a family carer of an adult with TBI (36); a RCT conducted in India, Pakistan, and Zambia examined early intervention in infants (38); a cross sectional study in Uganda focused on the child's cognitive ability (40) and a discussion piece from the authors of the only included study considered issues of implementation in supporting carers in LMIC (33). Our included paper utilised a RCT to a psychotherapeutic approach for reducing depression among carers in Uganda (31)**.**

### 3.5 Interventions

Four interventions from high income countries utilized online tools to improve executive functioning in adolescents with TBI (35), to improve psychological functioning in carers (24), and psychological functioning in those from low income backgrounds (37). One study employed an online information linking service for carers of children with developmental and epileptic encephalopathy which allowed carers to contact healthcare professionals with questions (39). All online interventions had some element of clinician involvement.

A cross sectional study from Uganda explored the use of meditational training to enhance carers sensitivity to their child (40). A case report suggested using medical and psychiatric social workers to provide psychosocial care to decrease carer burden and included preoperative counseling, carer education, resource mobilisation, enhancing social support and dealing with trauma reactions, stress, anxiety (36). A study conducted in three LMICs utilized The Partners for Learning (41) curriculum to introduce playful interactive learning activities modeled to carers during home visits (38). The intervention sought to improve children's outcomes in four core areas; cognitive and fine motor; social and self-help; gross motor; and language skills (38).

Our included paper sought to investigate the impact of group based interpersonal psychotherapy (ITP-G) compared to usual care (UC) in reducing depression in carers and children with nodding syndrome from 13 villages (ITP-G = 73 & UC = 69 carers) (31). Carers exhibited lower rates of depression at one and six months after receiving ITP-G compared to those receiving UC with a similar effect shown in children whose parents had received ITP-G (31). Studies from LMIC chose not to employ an online approach to deliver their interventions perhaps due to a lack of available technology, poor internet access and the associated costs.

### 3.6 Funding sources

Only one of the excluded studies was unfunded (36) and comprised a relatively low cost case report. Funding for the included paper came from awards based in Canada and the USA (31).

## 4 Discussion

This review has demonstrated a dearth of literature on interventions to support the mental health of carers of children with ABIs. Family carers of children with brain injuries face a range of challenges which may include family disruption and financial pressures, which impact on their quality of life and well-being (42, 43). Complex sociocultural experiences when caring for youth with disability are also routinely described by carers in LMIC, including shame about the child's condition, worry about being treated differently, and significant social stigma within their community (44). These stressors may result in psychological distress as the carers try to manage the day-to-day sequelae of their child's injury both acutely and in the longer term (19, 45). Support provided for children who have sustained brain injuries, needs to be extended to those caring for them (46). However, research shows that family needs (which includes carers’ needs) are often overlooked and thus, not addressed (19).

The lack of interventions for family carers is unfortunate given the wealth of existing literature which shows the benefits of support for carers of children with ABIs. For example, a recent systematic review (47) found that both in-person and remote interventions are effective in reducing depression, anxiety, psychological distress, and other psychiatric symptoms among carers of youth with brain injury. While most of the included studies were conducted in high income countries, they underscore the importance of family-centred rehabilitation interventions and services which extend to carers following paediatric brain injuries (19, 46). Interventions for family carers in contexts where rates of ABIs are elevated, such as in LMICS (48), are therefore even more necessary.

The included study, Mutamba et al. (31), sought to demonstrate the effectiveness of group based interpersonal psychotherapy (ITP-G) compared to usual care (UC) in reducing depression in carers of children with a specific form of ABI (nodding syndrome). The omission of an explanation of “usual care” did not impact on study quality due to a lack of assessment by the MMAT, yet this does impact the clarity of this work. Nodding syndrome is thought to be an infection-mediated illness characterized by new onset seizures and mental/physical deterioration (49). While relatively unique to Sub-Saharan Africa, immune-mediated diseases of the central nervous system in childhood are a major public health concern across LMICs (50), suggesting potential for the generalizability of this study's findings more broadly. Mutamba et al. (31), also aimed to investigate the impact of carer ITP-G on youth who participated in the study. While this addition is commendable, it potentially serves to exemplify the perspective that carers are often viewed as an important component of child outcomes, notwithstanding the importance of the outcome for carers themselves.

Given the international scope of this review, the identification of only one paper is surprising. This may lead to the conclusion of a lack of attention to the issue of mental health among family carers in LMIC. However, this outcome might also reflect contextual issues within LMICs more generally. Limited service delivery of mental health training and interventions, challenges to the implementation of interventions of this nature in these settings due to financial and resource constraints, and consequently, a lack of published research on the topic in such contexts, results in greater challenges to the implementation of such studies in LMICs (51). Hence the literature reflects a mismatch between need for mental health services and the service delivery thereof for carers of children with brain injuries in many LMICs. It is imperative that service providers and researchers devise programmes to better meet this population's need.

The excluded papers also provide good learning in that some sought to intervene in low-income regions of developed nations or with children in LMIC. The papers demonstrate the potential of utilizing medical or psychiatric social workers, as well as in-home and on-line activities to support carers who are dealing with trauma reactions, stress, and anxiety. One paper conducted with carers from low-income families in a developed nation sought to use an online intervention to increase access to information and specialty trained professionals at reduced costs (24). Notably, this study found that the online programme was particularly effective in reducing caregiver psychological distress in lower-income participants. However, the effectiveness of interventions developed in high-income countries is untested in LMIC. For example, individuals asked to take part in an online programme may struggle to afford the price of internet access or phone data. Moreover, authors have provided both equipment and internet access for their research e.g., Wade et al. (35), thus raising the question of sustainability following study completion.

### 4.1 Implications for policy and practice

The mental health needs of family carers of children and young people with brain injury in LMIC are not being taken into adequate consideration. Often, carers are seen as a means to support their children rather than people who are themselves in need. There is a broad literature base within developed countries documenting the bidirectional impact of parental distress on children's behavior and wellbeing, with this relationship holding strong even in the very early years of child development (52, 53). High levels of parenting stress adversely affect the general well-being and health of parents themselves, however, and is associated with a range of negative parenting practices including hostility (54), harsh discipline (55), and child maltreatment (56). Family carers support the work of clinicians in delivering care to children often without recognition of the stresses and strains they face. Service providers should consider the pivotal role of family carers in delivering care, rehabilitation, and supporting the prosocial functioning of their children with ABI and seek to better support them.

### 4.2 Strengths and limitations of this review

This scoping review followed the Preferred Reporting Items for Systematic Reviews and Meta-Analyses (PRISMA) guidelines (28) and the authors consulted with a specialist subject librarian to identify key search terms. Data extraction and quality appraisal were independently conducted by two reviewers using standardised data extraction and quality appraisal tool. However, due to resource constraints it was not possible to include articles published in languages other than English. Therefore, some important papers may have been missed. Additionally, it is possible that research articles were published using key terms unique to specific medical conditions and thus not included in the list of brain injury search terms.

### 4.3 Future research

Family carers in LMIC face significant pressures due to their child's disability and require bespoke interventions to improve their mental health. Interventions should be co-designed with family carers from LMIC to ensure any such programme adequately addresses their needs. Further, researchers designing interventions in high income countries should consider the generalisation of their findings and programmes to LMIC contexts. This might allow for existing and effective interventions to be adapted and introduced where they are most needed.

## 5 Conclusions

While there is a wealth of literature showing the benefits of support for carers of children with ABIs (57–59), challenges with complex cultural beliefs, legislation and policy, finances and resources, and consequently, a lack of published research on the topic have limited the implementation and availability of such resources in LMICs (44, 51). The results of this scoping review highlight the lack of focused research on the mental health of family carers of children with brain injuries in LMICs. It is imperative that service providers and researchers devise programmes to better meet this need.

## Data Availability

The raw data supporting the conclusions of this article will be made available by the authors, without undue reservation.
